# Reusable Component Model Development Approach for Parallel and Distributed Simulation

**DOI:** 10.1155/2014/696904

**Published:** 2014-03-03

**Authors:** Feng Zhu, Yiping Yao, Huilong Chen, Feng Yao

**Affiliations:** ^1^State Key Laboratory of High Performance Computing, National University of Defense Technology, Changsha 410073, China; ^2^College of Information System and Management, National University of Defense Technology, Changsha 410073, China

## Abstract

Model reuse is a key issue to be resolved in parallel and distributed simulation at present. However, component models built by different domain experts usually have diversiform interfaces, couple tightly, and bind with simulation platforms closely. As a result, they are difficult to be reused across different simulation platforms and applications. To address the problem, this paper first proposed a reusable component model framework. Based on this framework, then our reusable model development approach is elaborated, which contains two phases: (1) domain experts create simulation computational modules observing three principles to achieve their independence; (2) model developer encapsulates these simulation computational modules with six standard service interfaces to improve their reusability. The case study of a radar model indicates that the model developed using our approach has good reusability and it is easy to be used in different simulation platforms and applications.

## 1. Introduction

With the rapid development of simulation platforms and simulation applications during the last decades, in particular for parallel and distributed simulation, one of the most important challenges is how to respond quickly to new application requirements while reducing the development costs [1–3]. Building application from existing simulation models rather than from scratch is considered as a promising approach to improve the development efficiency, as well as to minimize engineering efforts and resource costs [4–6]. Reuse-oriented models are developed to be reused across simulation platforms with little or even no modification. At the same time, the reuse of component models together with visual programming technology makes it possible to drag and drop existing component models to assemble the simulation application, thus significantly reducing development time [7–9]. In addition, model reuse not only improves productivity but also has a positive impact on the quality of software products because of the obvious fact that a simulation application will work properly if it has already worked before [10, 11].

Motivated by the advantages of model reuse, various reusable model development approaches have been developed. (1) Based on a specific modeling language in which people designs special simulation function module as primitive and control module as simulator, different models created with the same modeling language can be reused for the corresponding simulator. However, most of simulation modeling languages are usually related to domain knowledge closely and each research field involves some specific platforms, so that there is congenitally deficiency for them to create models that can be reused across multidomain platforms, such as continue system simulation language *ACSL *[12], discrete event system simulation language *GPSS *[13], and multifield physical simulation language *Modelica *[14]. (2) Using a modeling specification which defines uniform internal structure, behavior constraint, and external interfaces for models, models will have good reusability if they do not bind with any platforms. However unfortunately, existing modeling specifications do not emphasize that model development should be independent with other simulation platforms. For example, *ESA* proposed a reusable simulation model description specification *SMP *(Simulation Model Portability Standards) [15, 16]. It is not supported well to reuse the models on other runtime platforms, because the execution of a *SMP* model depends on services provided by the SMP simulation platform. *The University of Arizona* gives a thorough discussion of parallel discrete event system specification *DEVS *[17], which mostly focuses on the hierarchical structure of components. (3) Some models are based on simulation environment which provides runtime supporting platform for model running. The reusability of them is also limited. Taking *HLA* [18] for instance, each federate provides some interfaces which compiles with the *HLA* interface specification, and federates developed by different developers can communicate with each other via the runtime infrastructure using these interfaces. But these federates are hard to use in other simulation platforms, such as *SUPE *[19], *POSE* [20], and *Charm++ *[21], because these platforms cannot “identify” any HLA service interfaces. In summary, as far as the authors know, there is not an adapted approach to guide users to develop models to achieve model reuse across platforms currently. Therefore, we have to identify a reliable way to develop what we mean by a “reusable” component model. Such an approach may help us not only to learn how to build reusable components but also to recognize whether a component model can be reused in a new simulation application.

In this paper, we first discussed a reusable component model framework composed of a simulation computational module and six well-defined standard interfaces. Next we proposed a reusable component model development approach, which contains two phases. In the first phase, the independent simulation computational modules are implemented under three principles. In the next phase, model developers encapsulated these simulation modules with the six standard interfaces to improve their reusability. Our final goal is to achieve flexible cross-platform reuse and fast composition of component model in parallel and distributed simulation.

Section 2 discusses the process of a reusable component model-based simulation application development.  Section 3 introduces a reusable component model framework and discusses the execution flow of six service interfaces.  Section 4 presents our reusable model development approach which elaborates the three principles that domain experts must observe and the encapsulation process.  Section 5 describes a case study in the field of military simulation, to create a reusable radar model which can be reused in air-defense and antimissile of naval vessel system. Finally, our conclusion will be made with an indication of the future work.

## 2. Reusable Component Model-Based Simulation Application

The term *reusable component model* refers to an independent replaceable part of a simulation application that can be independently developed and delivered as a unit and reused in different platforms [22]. Such component models can be selected from a model resource library, thus reducing both model developing time and costs when compared with a new development [23]. Meanwhile, reuse increases the reliability of the components, furnishing added “testing,” with the consequence that library component models are more reliable and less prone to faulty behavior [24].

**Figure 1:**
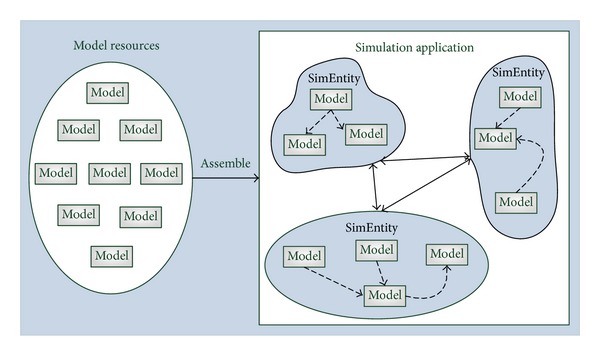
Component model-based simulation application development process in parallel and distributed simulation.

Component-based software development is associated with a shift from object-oriented coding to system building by plugging together components [25].  Figure 1 shows the simulation application development process based on reusable component models in parallel and distributed simulation. Component models are selected from a model resource library to construct simulation executable units, namely, simulation entities, which are used to assemble a simulation application running on the corresponding simulation platform. Unidirectional dotted lines between component models represent that simulation entity dispatches component models to compute according to the input and output relationship, while bidirectional active lines between simulation entities represent that simulation entities communicate with each other via external services provided by the corresponding simulation platform. Because simulation entities are different between various simulation platforms, if there are no constrains for model development, these models will be hard to reuse across different simulation platforms. As discussed earlier, models which call *HLA* interfaces are hard to be used in any other discrete event simulation platforms. Thus, to achieve model reuse not only in *HLA*, but also in many other simulation environments, we have to define a component model framework as a standard reusable model specification first.

## 3. Reusable Component Model Framework

Our reusable component model framework mainly contains a simulation computational module and six standard service interfaces. The simulation computational module is the internal implementation which contains a set of mathematical functions to model a process or function in real world. Six service interfaces, including state restoring interface, input interface, dynamic data driven interface, business process interface, state getting interface, and output interface, are used to encapsulate the internal mathematical functions provided by the simulation computational model. Thanks to the six standard interfaces, the reusable component model is very easy to be assembled or replaced rapidly, which will decrease software development time.

**Figure 2:**
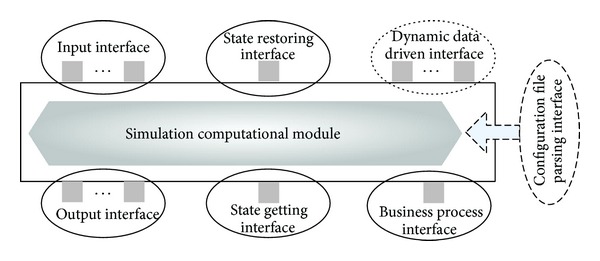
Reusable component model framework.

Figure 2 shows the reusable component model framework. As the figure shows, there is a configuration file parsing interface outside the simulation computational module. That is because most of component models have several attributes which need to be configured to satisfy the different demands for different simulation applications. Taking a radar model for example, we can assign different values for radar cross-section, probability of false alarm, or length of antennas to implement suitable radars for some specific applications. Moreover, our component model framework provides a state rolling back strategy, which makes the component model used in the simulation application with optimism mechanism. Different from many other methods storing the state of a model by itself, our strategy uses the state getting interface to get the model state data by the outside simulation entity. Then the simulation entity will define a roll back variable to save the state data, so no models need to do the “saving” thing. When rolling back happened, the simulation entity gets the value of state variable referent to the current simulation time, and then it calls the state restoring interface using the state variable as parameter to roll back the state of the reusable component model.

**Figure 3:**
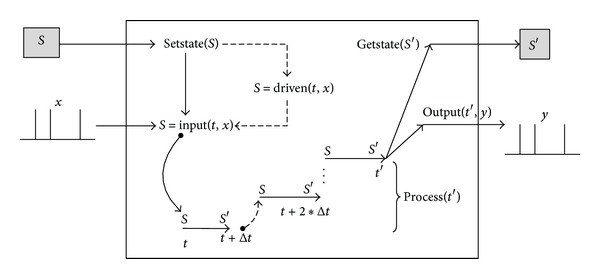
Execution flow of reusable component model.

Figure 3 shows the execution flow for a reusable component model, the details of which are described in the following.

1. Call state restoring interface *setstate* (*S*) to restore the state of the component model to *S*, which is a variable saving the state data of the simulation model. If this interface is called in the first time, *S* is on behalf of initial state data; otherwise, *S* is the state data stored by external simulation entity after once computation.
2. Call dynamic data driven interface *driven *  (*t*, *x*)  to provide dynamic data for the component model during its execution. *t* is the current time and *x* represents dynamic external input parameter set which depends on external simulation entity, not always needed for model computation each time.
3. Call input interface *input *  (*t*, *x*)  to provide input data for the component model during its execution. *t* is the current simulation time and *x *represents input parameter set which is always needed for model computation each time.
4. Call business process interface *process *  (*t*′)  to implement some important behaviors in a model of interest though internal state transition functions. After its execution, the local simulation time of the component model advances to *t*′.
5. Call output interface *output *  (*t*′, *y*)  to output the result after model processing. *t*′ is the current local simulation time and *x* represents an output parameter.
6. Call state getting interface *getstate *  (*S*′)  to get the state data of the component model after model processing and *S*′ represents the state data of the component model to be saved.

## 4. Two-Phase Development Approach

As discussed above, a component model can be considered as an implementation of a system, process, or function. It usually has a close relationship with multidisciplinary domain knowledge such as hydromechanics and aerodynamics, so that a software developer without domain expert's help cannot afford to create a model with high quality. On the other hand, if we know there are some component models that we can use again, probably with some slight modification, then we will do so as long as we trust the person who creates the model [26].

As lack of guideline for component model encapsulation, the models are usually characteristic of diversiform interfaces, coupled tightly, and bound together with simulation platforms. This implies that the integration of these models to a simulation application is a great challenge for developers, not to mention to reuse them.

**Figure 4:**
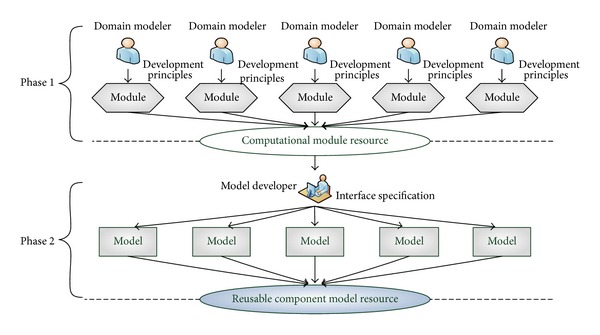
Two-phase reusable component model development process.

In view of the above reasons, our reusable model development approach contains two phases shown in Figure 4. In the first phase, domain modelers create a simulation computational module distributed observing three principles in the following, not only to reduce the overlap time of development, but also to improve its credibility. In the next phase, this simulation computational module is encapsulated to a reusable component model by a model developer according to the above six service interfaces to improve its reusability.

**Figure 5:**
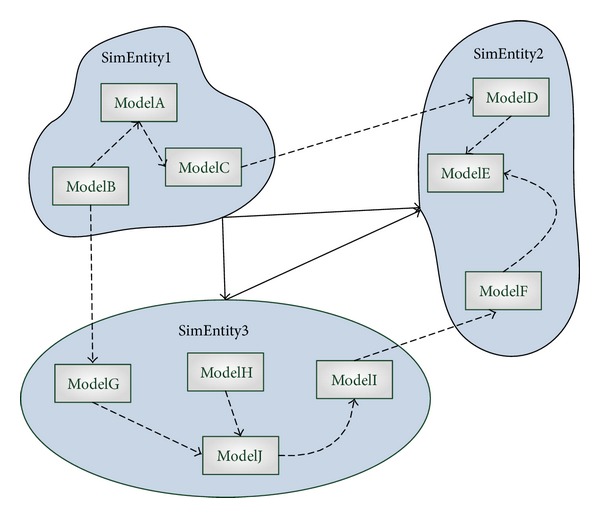
Communication between component models.

**Figure 6:**
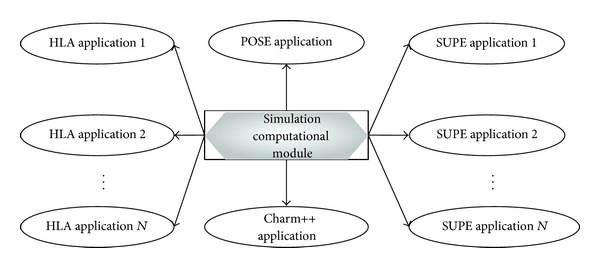
Model reuse across different platforms.

*Phase 1: Domain Modeler Implements Internal Simulation Computational Module*. According to component-based simulation application development paradigm, component models are to be reused across various products and product families. Since the internal state transmission functions are provided by the simulation computational module, to achieve model reuse across different platforms, the simulation computational module must be characterized and constrained properly. There are three principles that domain modeler must observe while creating simulation computational modules.

(*1) Simulation Computational Module Should Be Independent with Other Modules*. To achieve loose coupling and modularity, the execution of each module cannot depend on any other modules. This means that each module cannot call functions or access shared data in other modules. Modules which are developed by different domain modelers only communicate with each other via the external simulation entity. Taking Figure 5 for example, there are two situations according to whether two communicated models are in the same simulation entity or not. If the two models are in the same simulation entity, like *ModelA* and *ModelB*, *SimEntity1* will dispatch *ModelB* to compute first and then takes the output data of *ModelB* as the input data of *ModelA*. If the two models are in different simulation entity, like *ModelB* and *ModelG*, *SimEntity1* will also dispatch *ModelB* to compute first and then transfers the output data of *ModelB* to *SimEntity3*, which will receive the data and paper it as the input data of *ModelG*.

(*2) Simulation Computational Modules Should Be Independent with Simulation Platforms*. Simulation execution can be taken as several simulation computational modules running on a simulation platform. The simulation platform provides runtime environment for these computational modules. However, there are great differences between the mechanisms of different simulation platforms. For example, in Figure 6, in *HLA*, component models are used to construct federates which communicate with each other via *HLA* service interfaces provided by the runtime infrastructure, while, in discrete event simulation, component models are used to construct simulation objects which communicate with each other by calling event-scheduling interface provided by corresponding simulation engine. As a consequence, to achieve model reuse across different simulation platforms, the implementation of computational modules should be independent with simulation platforms; in other words it cannot call any service provided by any simulation platforms. If a component model calls *HLA* service interfaces directly, it is hard to be reused in other simulation platforms.

(*3) Simulation Computational Modules Should Provide Important Description Information for Users*. With the purpose of protecting intellectual property rights, modules are always encapsulated as a “black box” to model developers. The implication of this is that, unless a computational module is quite simple, a model developer will have to spend a great deal of time understanding how the computational module works. Thus, to facilitate model developers to encapsulate computational module, some important informal descriptions should be provided, which encompasses the following characteristics.

*Phase 2: Model Developers Encapsulate the Simulation Computational Module with Six Standard Service Interfaces*. The simulation computational module developed by a domain modeler has high reliability but pool usability. How to transform it to a component model with good reusability is our focus. Therefore on the precondition of no changes that happed for the business logic of the simulation computational module, model developers need to encapsulate the simulation computational module with six standard service interfaces in the following.

(*1) State Restoring Interface (int setstate(string & SimuState))*. This interface aims to restore the state of models to a storage state, where *SimuState* is a reference to a string parameter which contains the state data of the model related to the current simulation time. If the interface is called first, it means that *SimuState* represents the initial state data.

(*2) Input Interface (int input(InputDataTypeX inputData))*. This interface aims to provide input data for this model, where *InputDataTypeX(X=0, 1, 2 …)* is a user defined data type and *inputData* contains data of all the input variables needed to be assigned. Before calling this interface, the simulation entity applies enough memory for *inputData* to save data derived from another model output. The simulation entity usually needs to call this interface more than once for different input variables assignment because of various *InputDataTypeX*.

(*3) Dynamic Data Driven Interface (int driven(DynamicDataTypeX dynamicData))*. This interface aims to provide dynamic data for this model, where *DynamicDataTypeX(X=0, 1, 2,…)* is a user defined data type and *dynamicData* contains data of all the dynamic input variables needed to be assigned. We design this interface to support that a model usually needs to receive some unexpected data to amend its behavior under execution, such as adjusting radar cross-section or length of antennas for a radar model. Before calling this interface, the simulation entity applies enough memory for *dynamicData* to store data derived from outside environment. The simulation entity usually needs to call this interface more than once for different dynamic input variables assignment.

(*4) Business Process Interface (int process(double dSimuTime))*. This aims to call internal transmission functions provided by the simulation computational module to implement the business logic, where *dSimuTime* refers to the current simulation logical time.

(*5) Output Interface (int output(OutputDataType & ouputData))*. This interface aims to get output data from this model, where *outputDataTypeX(X=0, 1, 2,…)* is a user defined structure type and *ouputData* contains all of the output variables. Before calling this interface, the simulation entity needs to define a variable of *OutputDatatTypeX*. The simulation entity usually needs to call this interface more than once to get different output variables because of various *outputDataTypeX*.

(*6) State Getting Interface (int getstate(string & simuState))*. This interface aims to get the current state of simulation model, which will be stored in a string parameter, where *simuState* is a reference to a string parameter defined in the external simulation entity. After the component model execution finished, the simulation entity will use *simuState* as a parameter to call state restoring interface, where the state data of this model will be stored into parameter *simuState*.

After encapsulation finished, component models can be tested and executed in other projects. In order to facilitate component models reused across various products and product families, interface parameters of models will be added into model's informal descriptions, which with “domain,” “purpose,” and “usage time” together will be written into a model description file using XML technology to help users understanding how the component model works; see Table 1.

## 5. Case Study: A Reusable Radar Model

Model reusability appears to be a topic of notable interest in the simulation research community and in selected application areas [27], especially in military simulation. Radar was originally developed to satisfy the needs of the military for surveillance and weapon control [28]. In this section, we take the radar model under a complex electromagnetic environment, for example, to test and verify our reusable model development approach.

### 5.1. Create Simulation Computational Module

**Figure 7:**
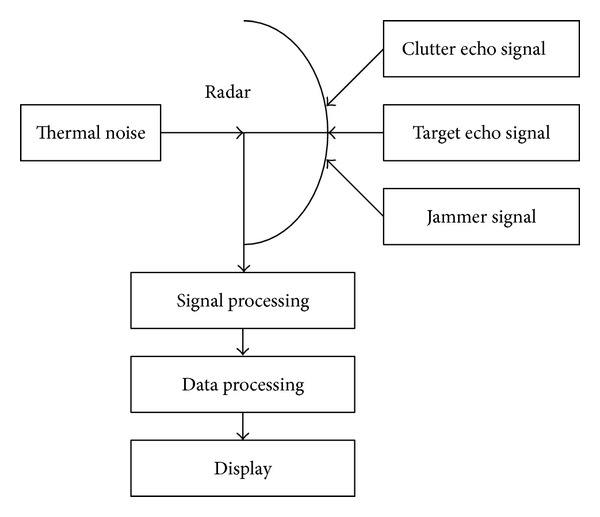
A radar model detection process.

The basic parts of a radar model in complex system simulation application are illustrated in the simple block diagram of Figure 7. The radar signal is generated by the transmitter and radiated into space by the antenna. Reflecting objects (targets) intercept and reradiate a portion of the radar signal, a small amount of which is returned in the direction of the radar. The returned echo signals which contain target echo signal, clutter echo signal, and interfering signal under complex electromagnetic environment are collected by the radar antenna. The thermal noise of radar itself will also be considered. According to the radar equation, after signal processing and data processing, we compute statistical probability of radar to detect a target. If the output of the radar receiver is sufficiently larger than a random variable, detection of a target is said to occur [29].

(1) The radar equation gives the range of a radar in terms of the radar characteristics, one form of which gives the received signal power as [29]

(1) $$\begin{array}{c} P_{rs}=\frac{P_{t}G_{t}}{4\pi R^{2}}\times \frac{\sigma }{4\pi R^{2}}\times \frac{G_{r}\lambda^{2}}{4\pi L}. \end{array}$$

The received signal powers are influenced by three factors to represent the physical process. The first factor is the power density at distance *R* meters from radar that radiates a peak power of *P*_*t*_ watts from an antenna of transmission gain *G*_*t*_. The value of the second factor is the target cross-section *σ* in square meters. The third parts contain antenna reception gain *G*_*r*_, radar wavelength *λ*, and radar comprehensive loss *L*.

(2) The receiver noise power generated by radar is described as [29]

(2) $$\begin{array}{c} P_{n}=kTB_{r}F_{n}. \end{array}$$

The thermal noise is equal to *kTB*_*r*_, where *k* is *Boltzmann's* constant, *T* = 290 K is the temperature (approximately room temperature), and *B*_*r*_ is receiver bandwidth. The receiver noise is the thermal noise multiplied by the factor *F*_*n*_.

(3) When the detection of the radar signal is influenced by an external noise source, such as a deliberate noise jammer, the receiver noise power is now that determined by the jammer (radar active interfering) rather than the receiver noise [30]

(3) $$\begin{array}{c} P_{rj}=\frac{P_{t}G_{t}}{4\pi R_{j}^{2}}\times \frac{B_{r}\lambda^{2}}{4\pi R_{j}^{2}}\times \frac{1}{B_{j}L_{j}}, \end{array}$$

where *R*_*j*_ = jammer range from radar, *B*_*j*_ = jammer bandwidth, *P*_*j*_ = jammer power, *G*_*j*_ = jammer antenna gain, *L*_*j*_ = jammer comprehensive loss.

(4) The interfering unwanted clutter echoes can severely limit the detect ability of the target, such as chaff interfering signal in military simulation. So the receiver passive interfering signal power is described as [31]

(4) $$\begin{array}{c} P_{rc}=\frac{P_{t}G_{t}G_{r}\lambda^{2}\sigma_{c}}{{\left(4\pi \right)}^{3}R_{c}^{4}L}, \end{array}$$

where *G*_*r*_ is chaff antenna gain, *σ*_*c*_ is chaff cross-section, and *R*_*c*_ is chaff range from radar.

(5) The minimum detectable signal can be expressed as the signal-to-noise ratio required for reliable detection times the receiver noise [32]

(5) $$\begin{array}{c} \text{SNR}=\frac{P_{rs}D_{j}}{P_{rj}+P_{rc}+P_{n}}. \end{array}$$

So in complex electromagnetic environment, the probability of radar to detect target can be written as

(6) $$\begin{array}{c} P_{d}={\left(\frac{\eta \text{SNR}+1}{\eta \text{SNR}}\right)}^{\eta -1}P_{f}^{1/(1+\text{SNR})}, \end{array}$$

where *D*_*j*_ is radar anti-interference factor, *η* is number of pulses, and *P*_*f*_ is the probability of a false alarm.

(6) Because radar equation generates statistical probability of detection, it needs a random variable to make decision. Supposing *μ* between [0, 1] is a random variable generated through Monte Carlo, if *μ* ≤ *P*_*d*_, we said that the target has been found.

We analyze the above mathematical formulas and transform them to the state transmission functions of the radar module in Table 2. According to parameter sources, we divided them into two categories. One is initialization parameter which is derived from the radar configure file, while the other is input parameter, which is derived from the output of other models.

### 5.2. Encapsulate with Service Interfaces

**Figure 8:**
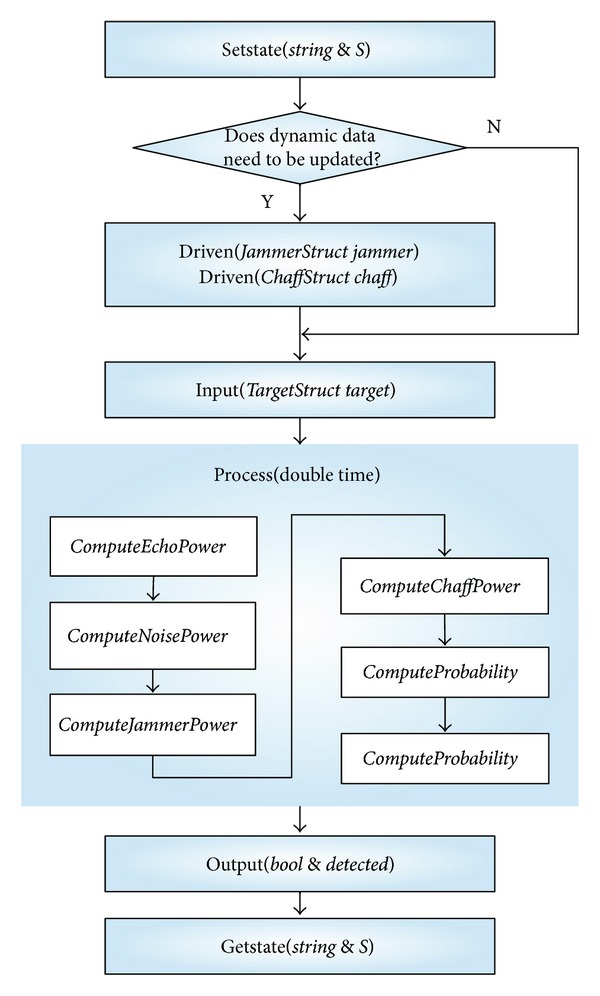
Reusable radar model execution flow.

According to the state transmission functions of radar module in Table 2, *ParseConfigure* provides access to radar configured file for external simulation entity. The dynamic data driven interfaces *driven (JammerStruct jammer)* and *driven (ChaffStruct chaff)* provide dynamic data for functions *ComputeJammerPower* and *ComputeChaffPower*. The input interface *input(TargetStruct target)* provides target data for function ComputeEchoPower. The business process interface will call *ComputeEchoPower*, *ComputeJammerPower*, *ComputeChaffPowe*, *ComputeProbability,* and *ComputeDetected* to implement behavior logic of the radar model according to the sequence recoding in the module informal descriptions. State restoring interface and state getting interface are utilized to implement state saving and restoring that make the component model used in simulation applications with optimism mechanism. Use state getting interface to get value of parameter *detected* that represents whether the target has been found or not. The execution flow of the radar reusable component model is described in the following; see Figure 8.

To illustrate the code structure of our reusable component model framework, we use C++ programming language for example to implement the radar component model shown in Algorithm 1.

## 6. Results and Discussion

Air-defense and antimissile of naval vessel is a typical scene in military simulation application [33, 34]. It usually contains three kinds of simulation entities: naval vessel, battle plane, and guided missile. The procedure of air-defense and antimissile of naval vessel is that, firstly, the battle plane opens its radar to search targets (naval vessel). Once it finds an enemy naval vessel, the battle plane will launch a guided missile to attack it; secondly, the guided missile flies to the enemy naval vessel under the control, and in the end of flight, it will opens its radar to locate the target by itself; finally, the naval vessel detects the guided missile using its radar and then uses jammer and interceptor missile for antimissile and air-defense.

**Figure 9:**
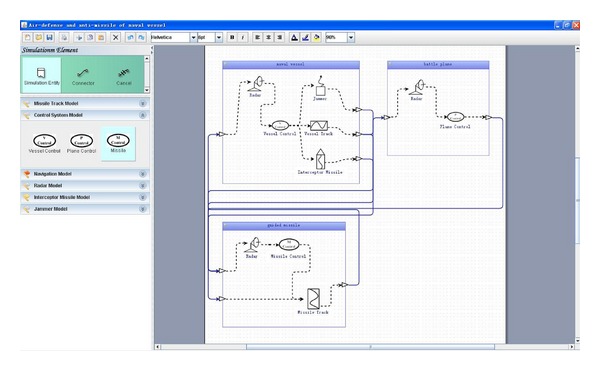
Snapshot of air-defense and antimissile of naval vessel simulation application.

**Figure 10:**
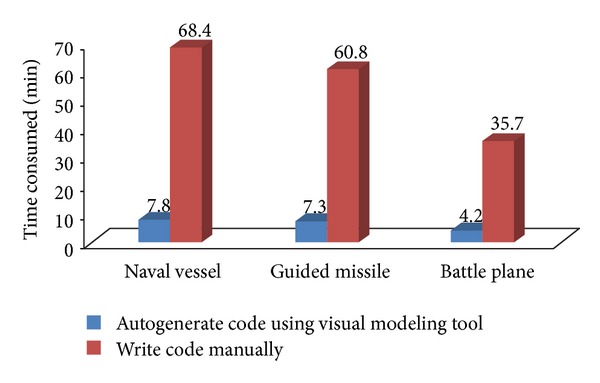
Time consumed of two approaches for developing three simulation entities.

Figure 9 shows the discrete event simulation application of air-defense and antimissile of naval vessel built with our visual modeling tool. A rectangular block represents a simulation entity and a small picture within the rectangular block represents a model in the right editing area. The component models, such as radar model, jammer model, and interceptor missile model, can be dragged and dropped from model resource panel on the left to construct simulation entities. They are connected with bidirectional active lines to build the air-defense and antimissile of naval vessel simulation application. Comparing with more than one hour cost through writing code manually, we only take less than 10 minutes to finish it using the visual modeling tool shown in Figure 10. It is only needed to move the mouse and the application code will be generated automatic in the background, thus significantly reducing development time.

We can see that each simulation entity, the naval vessel, the battle plane, or the guided missile, contains a radar component model. Although there are discrepancies between the radar component models in different simulation entities, maybe different wavelength, different transmission gain, or different interface parameters, it only needs to modify the configured file or adjust the external parameters to satisfy different demands rather than developing different models. For instance, while comparing the radar model in the guided missile within the naval vessel, the former has jammer and chaff data as parameters in its execution, but the latter has not. In this situation, the radar model in naval vessel will not call driven interface, and that is why we call it dynamic data driven interface. We can also assign different values for radar transmission gain or radar wavelength in the configure file to achieve suitable radars for the three kinds of simulation entities. Thus the same model can be used in different simulation entities that illustrates that the models developed using our approach have good reusability.

**Figure 11:**
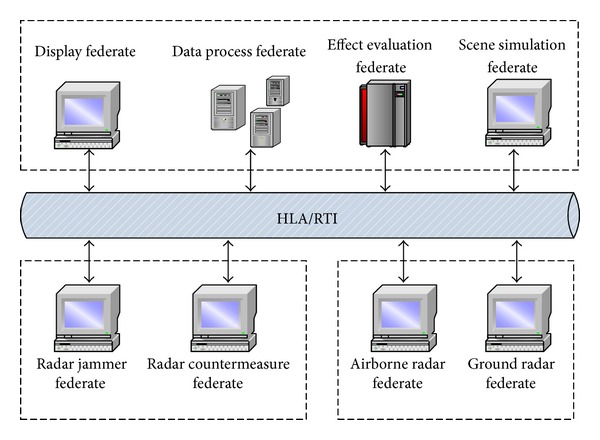
The architecture of a radar training simulation system.

Moreover, the radar model has been used in a training simulation system based on HLA [35, 36].  Figure 11 shows the architecture of the training simulation system. The airborne radar federate and the ground radar are the detection radars which are used to detect targets in two orientations. The radar countermeasure federate searches and parses the detection radar signal under complex electromagnetic environment. Based on the detection radar signal information, the radar jammer federate produces the external noise to interfere the two detection radar federates. The display federate, the data process federate, the effect evaluation federate, and the scene simulation federate are used to process data, display detecting result, and interfering effect.

In the radar countermeasure training simulation system, the airborne radar federate and the ground radar federate use the same radar model to implement their functionality. The practical project illustrates that the radar model is also very easy to be assembled in *HLA*-based distributed simulation system.

## 7. Conclusions and Future Work

Model reusability is very important for parallel and distributed simulation application development, not only for minimizing engineering efforts and resource costs, but also for improving the reliability. Current model development methods face several obstacles hindering model reuse across simulation platforms and simulation applications, such as diversiform interfaces, coupled tightly, and bound together with simulation platforms. To solve these problems, in this paper, we first presented a framework to define what we mean by “reusable” component model and then proposed a two-phase model development approach. In the first phase, the domain modeler builds a computational module that is independent with other models and simulation platforms. In the second phase, the model developer encapsulates the module with the framework as a model specification. At last, we tested our reusable component model development approach using a radar model and its application of air-defense and antimissile of naval vessel. Our method has been successfully applied in several projects. The case study and these practical applications illustrate that the simulation models created with our method have good reusability and facilitation to be assembled. They can also be used in different simulation platforms and applications.

As for our future work, we plan to study the algorithm for component models selection to suggest how to select component model to fulfill the requirements of different clients.

## Figures and Tables

**Algorithm 1:**
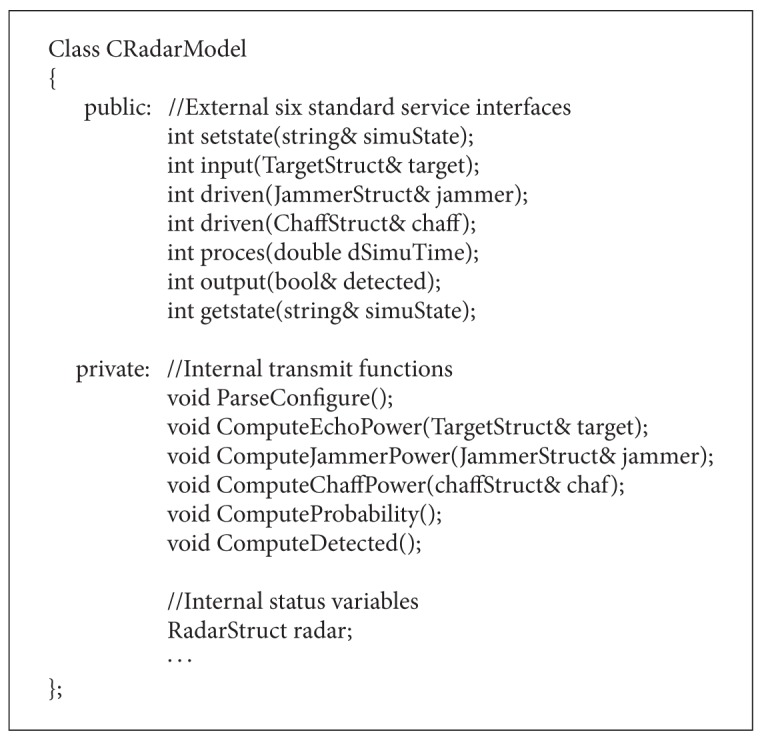
Reusable radar model code structure.

**Table 1:** Informal description of a computational module.

Domain	Application domains should be considered when characterizing the context for a component model, for example, hydromechanics, aerodynamics, mechanical control, and so on.

Purpose	Component models can be considered as replaceable building blocks of application. This includes the problem that the model solves.

Usage time	Usage time is a characteristic of a component that reflects its stability and maturity. When a component is first introduced, there is a high risk associated with its usage.

Parameter	This records the parameter description of the internal state transmission function, including name, type, unit, identification, and function, especially for user defined parameter types.

Sequence	The logical behavior of this simulation computational module is implemented though scheduling internal state transmission function according to correct sequence.

**Table 2:** Functions of radar computational module.

Function	Parameter	User defined type	Value
*ParseConfigure *	*P*_*t*_, *G*_*t*_, *λ*, *B*_*r*_, *F*_*n*_, *D*_*j*_, *η*, *P*_*f*_	*RadarStruct *	Radar configure file
*ComputeEchoPower *	*σ*, *R *	*TargetStruct *	Target output
*ComputeJammerPower *	*R*_*j*_, *B*_*j*_, *P*_*j*_, *G*_*j*_, *L*_*j*_	*JammerStruct *	External dynamic data
*ComputeChaffPower *	*B*_*r*_, *σ*_*c*_, *R*_*c*_	*ChaffStruct *	External dynamic data
*ComputeProbability *	*D*_*j*_, *η*, *P*_*f*_	—	Radar configure file
*ComputeDetected *	*Detected *	—	Radar output
